# Adipokine in Metabolic Liver Disease: Adipo–Brain–Liver Cross talk

**DOI:** 10.1155/ije/9009628

**Published:** 2026-08-05

**Authors:** Yule Su, Yiping Yang, Wenjuan Sun, Zhouxi Yu, Yilin Wang, Shixin Liu, Ziyi Chen, Yanfei Li, Xi Yang, Junyan Liu, Caikun Han, Yaqin Wang, Junhua Yuan

**Affiliations:** ^1^ Department of Special Medicine, School of Basic Medicine, Qingdao University, Qingdao, China, qdu.edu.cn; ^2^ Weight Management Center, Shenzhen Qianhai Shekou Free Trade Zone Hospital, Shenzhen, China; ^3^ School of Medicine, University of Health and Rehabilitation Sciences, Qingdao, China; ^4^ Department of Nephrology, Qingdao Municipal Hospital, Qingdao, China, qdslyy.cn

**Keywords:** adipokines, adipose tissue, brain, energy metabolism, nonalcoholic fatty liver disease

## Abstract

**Purpose:**

Adipokines play a key role in the interaction between the brain, liver, and adipose tissue. In this review, we selected adipokines with wide central receptor expression and summarized their indirect regulation on liver metabolism by acting on specific neurons and inducing neuropeptide production.

**Methods:**

A thorough PubMed search of peer‐reviewed studies was conducted using suitable keywords.

**Results:**

Since adipokine receptors could be expressed in both the brain and liver, adipokines could regulate energy homeostasis either directly through receptors expressed in the liver or indirectly by acting centrally. The latter can be achieved by acting in brain regions that have neural projections to the liver. Furthermore, growing evidence demonstrates that adipokines, while playing the above roles, are closely linked to the progression of nonalcoholic fatty liver disease (NAFLD).

**Conclusions:**

This review revealed that adipose tissue could be involved in regulating liver–brain cross talk during energy regulation and metabolism‐related diseases. In addition, we focused on the relationship between the mechanism of action of these adipokines and the progression of metabolic syndromes such as NAFLD and explored their potential as novel biomarkers and therapeutic targets for the treatment of metabolic diseases.

## 1. Introduction

**FIGURE 1:**
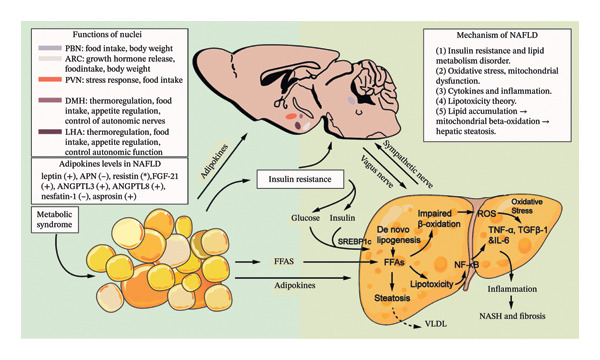
The link between adipose tissue, brain, and liver in NAFLD. Metabolic syndromes (obesity, hyperglycemia, dyslipidemia, etc.) result in abnormal mobilization of adipose tissue. Thus, an imbalance of free fatty acids (FFAs) and adipokines leads to hepatic fat deposition and steatosis. At the same time, ectopic accumulation of toxic metabolites produced by triglycerides can induce lipotoxicity and mitochondrial dysfunction. Certain adipokines could induce chronic inflammation and insulin resistance in the liver. In addition, adipokines can also act on certain nuclei in the brain to regulate lipid metabolism and inflammation in the liver by stimulating autonomic nerves. The above factors together affect the occurrence and development of NAFLD.

Recently, increasing evidence has supported the fact that adipose tissue should be considered an endocrine gland [1], since it could produce and secrete adipokines, a series of bioactive proteins/peptides and mediators, in an auto‐/paracrine manner, regulate important distant targets (such as the liver [2] and the brain [3]), maintaining metabolic homeostasis. Since the discovery of adipokines, studies have revealed their central roles in the cross talk network among key organs [4]. Conversely, the dysfunction of adipose tissue and dysregulation of adipokines are causally associated with metabolic disorders, including obesity [5], diabetes [6], nonalcoholic fatty liver disease (NAFLD) [7], and even cancer [8]. As a condition closely associated with metabolic syndrome, NAFLD has a very high and growing prevalence, with approximately one in every three people worldwide affected. NAFLD is not a single disease but a spectrum of progressive disorders. It typically starts with simple steatosis and gradually progresses to nonalcoholic steatohepatitis and cirrhosis [9]. Moreover, population‐based studies have demonstrated that NAFLD is the most typical underlying etiologic risk factor for hepatocellular carcinoma (HCC) [10]. The liver plays an important role in regulating whole‐body energy homeostasis [11], as it serves as the central metabolic hub for lipid metabolism and has a high capacity for fat accumulation. Excess energy contributes to adipocyte hyperplasia and hypertrophy, eventually leading to adipocyte dysfunction and death [12]. Under such metabolic stress, many adipokines are released, which act directly or indirectly on the liver, and affect its function (Figure 1).

Leptin, a widely distributed adipokine, could act on the liver and cause chronic inflammation, manifested as hepatocyte steatosis and liver fibrosis, which can eventually develop into NAFLD [13]. A recent randomized controlled trial reported that leptin reduced hepatic lipid content in humans via a vagal mechanism, indicating that adipokines might have antisteatotic properties that are independent of food intake by stimulating hepatic lipid metabolism via a brain–liver axis [14]. In addition, some adipokines, such as resistin and leptin, could act on the liver to affect glucose metabolism. Resistin plays a direct role in insulin resistance (IR) and induces Type 2 diabetes [15]. Adipokine‐induced IR leads to fat accumulation and hepatic immune responses, ultimately promoting the development of NAFLD [16]. A recent study showed that hypothalamic resistin action has a significant effect on endogenous glucose production and proinflammatory cytokine expression in the liver, suggesting that adipokines may also regulate hepatic glucose metabolism and influence NAFLD progression through the liver–brain axis [17]. However, few reviews have focused on this specific topic, and further research is needed to draw solid conclusions.

Some adipokines (e.g., nesfatin‐1) could act as interorgan messengers and cross the blood–brain barrier to the target brain area bidirectionally without saturation limits. The receptors of adipokines are widely expressed in the brain, as they play roles in maintaining energy homeostasis and lipid metabolism balance by acting on specific neurons and inducing the generation of neuropeptides [18]. Our previous studies suggested that adipokines (such as nesfatin‐1 and angiopoietin‐like protein‐8 [ANGPTL8]) act on specific nuclei of the forebrain [19], hypothalamus [20], and hindbrain [21–23] to affect appetite, food intake, and energy metabolism levels via altering the excitability of glucose‐sensitive neurons or through complex neural circuits. Interestingly, the target nuclei of adipokines were found to be located in neural projections directly to the liver. Moreover, signals generated by adipokine receptors in the brain can be transmitted to peripheral tissues and organs through sympathetic and parasympathetic nerves [24, 25]. Meanwhile, adipokines produced by metabolic disorders in peripheral organs can also be transmitted to specific brain regions through sympathetic or parasympathetic nerves, which in turn regulate the function of peripheral organs in a feedback manner. Our recent work showed that nesfatin‐1 intracerebroventricular (i.c.v.) administration activated lipid mobilization in adipose tissue via the sympathetic nervous system [26]. Furthermore, we found that nesfatin‐1 injection into the VMH promoted uncoupling protein 1 (UCP‐1) protein expression levels in white adipose tissue (WAT) and brown adipose tissue (BAT) via the β3 receptor in the hypothalamic–sympathetic pathway in mice [27]. Nesfatin‐1 also plays an important role in the occurrence and development of NAFLD. Research indicates that serum nesfatin‐1 levels in NAFLD patients were lower than those in healthy controls [28, 29], indicating that adipokines may play an important role in the central nervous system (CNS) regulation of peripheral metabolism. The liver is innervated by both the sympathetic and parasympathetic nerve systems. Various physiological parameters, including osmolality and metabolite (mainly glucose and lipid) levels, could be reported to the CNS, while a wide range of signals also come down from the CNS to the liver, regulating blood perfusion and energy metabolism [24]. However, no studies have reported the mapping of brain‐activating, liver–brain cross talking primary adipokines. Although substantial efforts have been dedicated to identifying novel adipokines, the precise mechanisms driving their secretion are still not fully elucidated, especially regarding the neuroendocrine dimensions of the adipo–brain–liver axis.

In the current review, the roles of adipokines in obesity‐related liver diseases were discussed in depth, with a specific emphasis on the cross talk among the adipose tissue, brain, and liver. Potential new models of neuroendocrine cues in the brain–liver axis were also reviewed.

Sex is an important biological variable in adipokine biology and in the progression of metabolic disease. Circulating leptin levels are generally higher in women than in men at comparable levels of adiposity, and adiponectin (APN) also shows clear sex‐related differences in humans [30, 31]. In addition, emerging evidence suggests that FGF21 secretion and metabolic responses may be sex‐biased under physiological and pathological conditions [32]. Sex differences in the adipose–brain–liver axis may reflect differences in fat distribution, sex hormone signaling, and adipokine sensitivity. Women generally have more subcutaneous fat and higher leptin and adiponectin levels, whereas men more often accumulate visceral fat, which is linked to inflammation and hepatic IR. These factors may contribute to sex‐specific differences in NAFLD susceptibility and progression [33–35].

In this review, we focused on seven adipokines, including leptin, APN, FGF21, nesfatin‐1, ANGPTLs, resistin, and asprosin. These molecules were chosen as representative mediators to characterize interorgan cross talk driving NAFLD pathogenesis and progression, given that they are either classic adipose‐derived factors or tightly linked to adipose endocrine function. Growing evidence indicates that these adipokines engage in the adipose–brain–liver axis via central receptor signaling, neuroendocrine modulation, and metabolic communication between peripheral tissues and the CNS, while also participating in the regulation of appetite, energy homeostasis, insulin sensitivity, lipid metabolism, and the development of metabolic disorders, especially NAFLD.

## 2. Leptin

Leptin, a polypeptide hormone composed of 167 amino acids encoded by the ob gene [35], is an adipokine discovered and named by Professor Friedman of Rockefeller University in 1994 [36]. Leptin is mainly synthesized by adipocytes from WAT; thus, the circulating levels of leptin in the body are proportional to the total energy stored as fat [37]. Leptin receptor (LEP‐R) is widely expressed across various tissue types, e.g., brain, adipose tissue, live, kidney, and muscle [35]. Nonetheless, the primary effects of leptin are exerted by specific neurons in brain, especially hypothalamus. It plays important roles in energy balance regulation, appetite reduction [38], metabolism promotion [39], and blood glucose regulation [40] (Table 1).

**TABLE 1:** Overview of adipokines, including their secretion sites, target tissues, genetic characteristics, targeted molecules, functional properties, associated pathological disorders, administration effects, and central signaling cascades.

Adipokines	Site of secretion	Target site	Genetic characteristics	Targeting molecule	Functional characteristics	Altered anomalies	Administration effect	Central pathway
Adiponectin	Adipose, endothelial cells, cardiomyocytes, muscle cells, and brain	Liver, endothelial cells, SkM, lateral hypothalamus, and Mehnert’s basal nucleus	Chromosome 3q27 APM1	AMPK, GLUT4, PPARα, PGC‐1α, IRS‐1, IL1‐β, TNF‐α	‐ Fat oxidation (+) ‐ Angiogenesis and protecting vascular wall (+) ‐ Gluconeogenesis (−) ‐ Mediate nitric oxide (+) ‐ Anti‐inflammatory ‐ Anti‐insulin resistance ‐ Antifibrosis ‐ Antitumor	‐ In NAFLD patient↓ ‐ In T2DM↓	‐ Weight and amelioration of InR in obese men↑ ‐ Blood glucose, insulin levels and hepatic gluconeogenesis in high‐fat, high‐sucrose (HFHS)–fed mice↓	‐ AMPK and PPARα pathway ‐ PI3K/Akt pathway ‐Through the activation of IRS1/2, ERK, Akt, FOXO1, JAK2 and STAT3

Leptin	Adipose, placenta, gastrointestinal tract, liver, ovary, skeletal muscle, pancreas, and brain	Hypothalamus, pituitary gland, heart, kidney, adipose	Chromosome 7 (7q31.3)	STAT3, STAT5, SOCS3, SHP2, MAPK, ERK, PI3K, AMPK	‐ Appetite (−) ‐ Energy expenditure (+) ‐ Fat synthesis and storage (−) ‐ Regulate energy homeostasis	‐ In NAFLD patient and obese people↓ ‐ In lipodystrophy syndromes patient↓ ‐ In patients with cardiovascular disease↑	‐ Food intake in human and rodents↓ ‐ Hepatic glucose production↓ ‐ The production of adipose tissue↓	‐ LEP‐Rb/JAK2/STAT3 pathway ‐ LEP‐Rb/RS/PI3K pathway ‐ LEP‐Rb/SHP2/MAPK (ERK1/2) pathway ‐ LEP‐Rb/AMPK/ACC pathway

Nesfatin‐1	Gastric X/A‐like cells and brain	Hypothalamus, midbrain, brainstem, esophagus, stomach, colon, small intestine, pancreas, liver, testes, adipocytes, cardiomyocytes, uterus, ovaries, epididymis	NUCB2 gene	AMPK, TORC2, STAT3, mTOR, PPARg, SREBP1	‐ Appetite (−) ‐ Energy expenditure (+) ‐ Fat synthesis and storage (−) ‐ Lipogenesis in adipocytes (−) ‐ Insulin resistance (−) ‐ Regulate energy homeostasis	‐ In NAFLD patient and obese people↓ ‐ In T2DM↓	‐ Food intake in human and rodents↓ ‐ Hepatic glucose production↓ ‐ The production of adipose tissue↓	‐ InsR/IRS‐1/AMPK/Akt/TORC2 pathway ‐ Downregulation of the adipogenic transcription factors PPARg and SREBP1 ‐ mTOR‐STAT3 signaling pathway

Asprosin	White adipose tissue	Liver, hypothalamus, testis, kidney, pancreas, skeletal muscle, heart	Chromosome 15q21.1	G protein, cAMP, PKA, GLUT4, AMPK, UCP1, Nrf2	‐ The release of glucose in the liver (+) ‐ Apoptosis and ROS generation (−) ‐ Insulin resistance (+) ‐ Type II diabetes and metabolic syndrome therapeutically (+) ‐ Appetite (+) ‐ Metabolic disorders (−)	‐ In NAFLD patient↑ ‐ in T2DM↑	‐ The insulin deficiency (−) ‐ Atherosclerotic burden↓ ‐ Skeletal muscle insulin sensitivity impairment	G protein‐cAMP‐PKA pathway in the liver

FGF21	Liver, adipose, pancreas and skeletal muscle	Hypothalamus, liver, heart, kidney, muscle, vessel, WAT, BAT and pancreas	Chromosome 19 (19q13.33)	β‐Klotho, AKT, ERK1/2, PPARγ, PGC1‐α	‐ Hepatic triglyceride↓ ‐ Insulin sensitivity of adipose tissue (+) ‐ Regulate energy homeostasis	‐ FGF21 level in association with mitochondrial myopathies↑ ‐ Serum FGF21 levels in overweight human↓	‐ Liver: lipid accumulation↓, NAFLD and NASH↓, VLDL‐TG secretion↓, fatty acid oxidation↑, gluconeogenesis↑ ‐ WAT: glucose uptake↑, lipolysis↑, thermogenesis↑, NEFA uptake↑ ‐ Brain: circadian behavior↑, hypothalamic pituitary adrenal axis↑	‐ Extracellular signal–regulated kinase 1 and 2 (ERK1/2) ‐ AMPK‐SIRT1 pathway

Resistin	Adipose, monocyte/macrophage, brain, WAT, peripheral blood, and liver	Liver, hypothalamus, and pituitary gland	Chromosome 19p13.3	TNF‐å,IL‐6, IL‐ß, SREBP‐1c, NF‐κB, SOCS‐3, AMPK	‐ Proinflammatory properties ‐ Cytokine signaling inhibitors↑ ‐ Amount and activity of mitochondria (−) ‐ Inflammatory response of the liver and the degree of fibrosis (+) ‐ Release of norepinephrine and dopamine from the hypothalamic synaptosomes↑	‐ Hepatic/adipose insulin resistance (+) ‐ Liver steatosis (+) ‐ Affect hepatic glucose homeostasis ‐ Hepatic glycogenesis (+) ‐ The amount and activity of mitochondria (−)	‐ IR (+) ‐ Hepatic glucose production and glycemia↑ ‐ mRNA levels of in the liver after systemically infused↑	‐ Protein kinase C/protein kinase G/P65/PPARC‐1α signaling pathway ‐ AMPK/PGC‐1å pathway ‐ Inhibit insulin signaling IRS‐1/PI‐3K/AKT pathway, hypothalamic–pituitary tract

Angptl3	Liver and brain	Liver and blood vessel	Angptl3 gene	LPL	‐ Plasma triglyceride↑ ‐ VLDL‐TG in WAT↑	‐ In NAFLD, T2DM, obesity patient↑	‐ Insulin sensitivity↑	‐ LPL—lipid uptake of hypothalamic neurons—brain lipid sensing↑

Angptl4	Liver and adipose	Blood vessel and adipocyte	Angptl4 gene	LPL	‐ Adipocyte lipolysis (+) ‐ plasma TG↑	‐ In obesity patient↑	‐ Inflammation↓ ‐ The clearance of lipid remnant particles↑	N/A

Angptl6	Liver and adipose	BAT and liver	Angptl6 gene	Leptin, ERK/MAPK, FOXO1	‐ Plasma FGF21↑ ‐ Mitochondrial oxygen consumption↑ ‐ Hepatic gluconeogenesis↓ ‐ Insulin resistance (−)	‐ In NAFLD, T2DM, obesity patient↑	‐ Hepatic gluconeogenesis↓	N/A

Angptl8	Liver, Adipose, and Brain	Blood vessel and liver	C19orf80 gene	AKT, IKKγ, SREBP1, PirB, NF‐kB	‐ Hepatic gluconeogenesis↓ ‐ Glucose tolerance (+) ‐ Insulin sensitivity (+) ‐ Lipogenesis (+) ‐ Inflammation (−)	‐ In NAFLD, T2DM, obesity patient↑	‐ Improve liver glucose and lipid metabolism homeostasis	‐ DMH—NPY—appetite↓

Leptin acts by binding to its receptors (LEP‐R). There are six known LEP‐Rs: LEP‐Ra, b, c, d, e, and f. Among these receptors, LEP‐Rb plays a major role [41]. Following the binding of leptin to LEP‐R, multiple signaling pathways are activated (Table 1): (1) LEP‐Rb/JAK2/STAT3 pathway—leptin binds to LEP‐Rb, intracellular signaling is initiated, and JAK2 phosphorylation/activation is allowed, followed by the phosphorylation of three tyrosine residues (Tyr985, Tyr1077, and Tyr1138) located in the intracellular domain of LEP‐Rb by JAK2. Among them, Tyr1138 mediates the activation of STAT3 [42, 43]. In the hypothalamus, STAT3 binds to the promoters of proopiomelanocortin (POMC) and AgRP, enhancing POMC expression, while suppressing AgRP and NPY expression, ultimately leading to reduced food intake [44]. (2) LEP‐Rb/IRS/PI3K pathway: The regulation of food intake heavily depends on the activity of the PI3K signaling pathway in the brain. Leptin binds to the hypothalamic LEP‐R, and Tyr1138 is phosphorylated, which subsequently activates STAT3. Activated STAT3 induces enhanced PI3K (phosphatidylinositol 3‐kinase) activity associated with IRS2. Leptin‐induced activation of PI3K in the hypothalamus has been shown to recruit phosphodiesterase 3B (PDE3), which inhibits cAMP activity, induces cAMP clearance, and functions to reduce food intake [45]. (3) LEP‐Rb/SHP2/MAPK (ERK1/2) pathway: Leptin binds to LEP‐Rb and initiates intracellular signaling to promote JAK2 phosphorylation. Tyr985 is subsequently phosphorylated by JAK2. The activated Tyr985 induced the activation of downstream signaling molecules such as SHP2 and MAPK. SHP2 downregulates the LEP‐Rb‐STAT3 pathway while promoting the activation of extracellular kinases regulated by leptin. Overall, SHP2 is a leptin signal enhancer, pharmacological enhancement of Shp2 activity may represent a novel approach worth considering in the clinical treatment of leptin resistance and obesity [46]. In addition, SHP2 participates in energy homeostasis maintenance. However, the functional activation of the abovementioned leptin occurs under physiological conditions. In pathological states, this signaling pathway undergoes a functional switch in the biological state due to leptin resistance or indirectly resulting in leptin resistance, manifesting as leptin functional loss. For instance, the increase of adipose tissue or metabolic disorders often lead to the increase in leptin secretion, resulting in leptin resistance [47] and the inhibition of the above three signaling pathways. In the liver, STAT3 activation leads to enhanced expression of SOCS‐3, a cytokine signaling suppressor, resulting in insulin and leptin resistance. At the same time, high leptin levels may promote the production of proinflammatory cytokines and induce the occurrence of liver inflammation [48]. Finally, it promotes the occurrence and development of NAFLD (Figure 1). In summary, to reconcile the seemingly contradictory dual roles of leptin in hepatic metabolism, leptin resistance must be emphasized as a central factor. Under physiological conditions, leptin acts centrally to reduce food intake and inhibit hepatic lipogenesis via the vagus nerve, thereby decreasing hepatic lipid accumulation. However, in obesity and metabolic disorders, central leptin resistance develops, which impairs the anorexigenic and insulin‐sensitizing effects of leptin. Under pathological conditions such as obesity and NAFLD, leptin resistance is further exacerbated by impaired transport of leptin across the blood–brain barrier into the CNS. This defect reduces central leptin sensitivity, leading to dysregulated energy homeostasis and peripheral metabolic disorders like IR and hepatic steatosis [49, 50]. Meanwhile, hyperleptinemia associated with leptin resistance promotes proinflammatory and profibrotic responses in peripheral tissues including the liver. Thus, the protective effects of leptin are mostly observed with pharmacological leptin supplementation or in leptin‐deficient models, whereas its detrimental effects predominate under leptin‐resistant conditions. Differences in central leptin sensitivity, intervention dosage, and disease status may account for the divergent outcomes reported in the literature.

Leptin acts on certain nuclei in the brain to activate neurons. Signal communications between the liver and key brain nuclei are mainly performed via sympathetic and parasympathetic fibers. While leptin serves as a key signal molecule. (1) Arcuate nucleus (ARC): ARC contains the majority of neurons that directly respond to leptin stimulation [51]. There are two types of neurons in the ARC: agouti‐related peptide/neuropeptide Y (AgRP/NPY neuron) and proopiomelanocortin‐/cocaine‐ and amphetamine‐regulated transcript neuron (POMC/CART neuron). AgRP/NPY and POMC/CART neurons can project to the PVN, VMH, DMH, and LHA [52]. Leptin can activate POMC/CART neurons while inhibiting AgRP/NPY neurons [51]. Excited POMC neurons release the appetite‐suppressing polypeptide α‐melanocortin‐releasing hormone (α‐MSH). α‐MSH enhances the activity of downstream melanocortin receptor 4 (MCR4) expressing neurons and reduces feeding and facilitates acute body weight loss [53]. In addition, the excitation of POMC neurons induces systemic sympathetic excitation [54, 55], which in turn promotes WAT fat mobilization, reduces hepatic triglyceride content [56], and increases hepatic glucose output by the rapid activation of glycogen phosphorylase, leading to a significant reduction in the intrahepatic glycogen content [57]. Chronic hepatic sympathetic overactivation also mediates hepatic steatosis and induces the development of NAFLD [58]. This could potentially explain why leptin may act as an inflammatory factor to further promote NAFLD progression. (2) Dorsal vagal complex (DVC): The DVC consists of two nuclei: nucleus tractus solitarius (NTS) and dorsal motor nucleus (DMN) of the vagus. DVC astrocytes functionally express the LEP‐R, mediating the anorexigenic effects of leptin [59]. Leptin‐induced activation of the vagus nerve in the DMN serves as a primary mechanism for modulating peripheral immune responses and cytokine release [60]. This activation enables the vagus nerve to directly diminish LPS‐induced TNF production in the liver and bloodstream. At the cellular level, binding of acetylcholine (Ach) released from vagal efferent nerve terminals could inhibit TNF production from macrophages and thus exert anti‐inflammation effects in the liver [61]. There is also research reporting that the vagus nerve α7nAChR‐mediated cholinergic signals have hepatoprotective antiapoptotic effects [62]. In addition, changes in the levels of cytokines and other inflammatory molecules in the liver can be detected in afferent vagus peripheral terminals and communicated to NTS [63] and there are neural projections between NTS and DMN [64] (Figure S1).

In conclusion, leptin plays a crucial role in maintaining energy homeostasis, suppressing appetite, and regulating blood glucose balance. It also influences liver inflammation and fat deposition by mediating the interaction between the liver and the brain. In animal models, there is sufficient evidence indicating that leptin is closely related to the occurrence and development of NAFLD. Its role in humans still requires further in‐depth exploration [65]. From a pathological perspective, leptin participates in regulating insulin sensitivity, lipid metabolism and lipid toxicity, oxidative stress response, inflammation activation, and fibrosis progression. These processes jointly drive the evolution of NAFLD from simple fatty liver to NASH and further develop into liver fibrosis, cirrhosis, and even HCC. Currently, the formation mechanism of leptin resistance and its dynamic role in different disease stages of NAFLD still need to be systematically clarified, and more research is needed to explore intervention strategies targeting the leptin pathway.

## 3. APN

**FIGURE 2:**
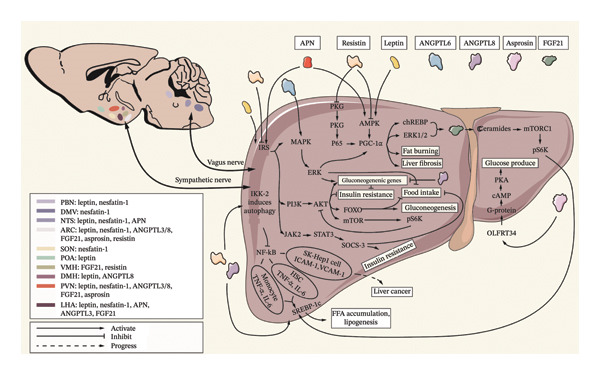
Various adipokines mediate the dialogue between the liver and brain. Adipokines can act in both the central nervous system and liver, with target nuclei present in neural projections that directly innervate the liver. The signals generated by these receptors can be transmitted to the liver via sympathetic and parasympathetic/vagal nerves to regulate hepatic metabolism. Adipokines exert their biological effects through activation of different or overlapping signaling pathways that lead to downstream cascades. Asprosin promotes gluconeogenesis via the G protein–coupled receptor‐mediated cAMP/PKA pathway. Resistin and leptin have opposing effects. Adiponectin, leptin, and FGF21 exert significant effects on biological processes such as insulin resistance through the PI3K/AKT pathway. Additionally, adiponectin, leptin, and resistin affect peroxisome proliferator–activated receptor γ coactivator 1α (PGC‐1α) to promote lipolysis in adipose tissue and liver fibrosis. Furthermore, ANGPTL8 induces FFA accumulation through the NF‐κB pathway, promoting lipogenesis or hepatocarcinogenesis.

APN was officially named by Arita et al. in 1999, which is a bioactive peptide or protein encoded by the APM1 gene containing 247 amino acid residues [66, 67]. APN is primarily secreted by adipocytes, yet it can also be synthesized by cardiomyocytes, endothelial cells, and muscle cells [68–70]. The receptors of APN include adipoR1 (ubiquitously expressed), adipoR2 (exclusively in the liver) and T‐cadherin‐CDH13 [71, 72], while the main biological effects are mediated by adipoR1 and adipoR2. In the CNS, the receptors are broadly expressed throughout forebrain (such as hippocampus) [73], midbrain (such as hypothalamus), and hindbrain (such as brainstem) [74–77] (Figure 2). However, the specific roles of these receptors in the brain disease and the mechanisms involved in cross talk between organs remain unclear (Table 1).

Central APN reduced food intake [78, 79]. Through the activation of IRS1/2, ERK, Akt, FOXO1, JAK2, and STAT3 mediated by adipoR1. However, Qi et al. reported that APN i.c.v. injection could enhance energy consumption without affecting feeding behavior [80]. Inhibiting food intake can reduce the intake of fat and fructose. Excessive intake of fat and fructose is a high‐risk factor for the occurrence of NAFLD/NASH [81]. Peripheral administration of APN had no obvious impacts on food intake but could reduce weight by enhancing fatty acid oxidation in both skeletal muscle and liver [82–84]. Reducing weight can reduce lipotoxicity and inhibit inflammatory pathways, thereby improving the pathophysiology of NAFLD/NASH [85]. Moreover, the negative correlation between plasma APN and leptin levels/body weight leads to the hypothesis that APN might play a central role in energy homeostasis. Although APN mRNA is undetectable in human brain extracts, adiponectin and its receptors are expressed in the pituitary and selected brain regions, indicating a local neuroendocrine regulatory system rather than a purely circulating endocrine mode of action [77]. In the CNS, AdipoR1‐ and AdipoR2‐mediated signaling may modulate AMPK and PPARα‐related pathways, influence hypothalamic energy homeostasis, and regulate BBB endothelial inflammatory signaling [86]. Moreover, the selective presence of trimeric and hexameric adiponectin in cerebrospinal fluid (CSF) suggests that CNS access is restricted to specific oligomeric forms, which may be sufficient for receptor activation in defined neural niches. Therefore, the central effects of adiponectin are likely mediated through a combination of pituitary paracrine/autocrine signaling, hypothalamic neuronal circuits, and neurovascular regulation, which may secondarily improve hepatic glucose and lipid metabolism via the hypothalamus–autonomic nerve–liver axis [87] (Table 1) (Figure 2).

Among the various adipocyte‐derived hormones that act in CNS, adiponectin is an important one. It had been reported that adiponectin could regulate autonomic function, energy homeostasis, and various organ physiological states [88]. The expression pattern of adiponectin in vagal afferent pathways had been explored by Kentish et al. who detected adiponectin receptor expression in the nodose ganglia in mice, where a modulatory role of adiponectin had been revealed toward gastric vagal afferent satiety signals under metabolic stress [89]. Additionally, adiponectin receptor expression was also reported in the NTS by Hoyda et al. [90], and the latter is the major viscerosensory integration site. Moreover, the microinjection of APN in the medial NTS influenced single neuron function and blood pressure, indicating that NTS in the hindbrain was a new CNS site other than the generally accepted hypothalamus, upon which APN may act to influence central autonomic processing [91–93]. Park et al. injected recombinant adiponectin into the lateral ventricle of rats and found that it led to a reduction in visceral fat due to increased energy consumption and fatty acid oxidation. Additionally, intraventricular injection of adiponectin directly acted on the liver and peripheral tissues through the autonomic nervous system (especially the sympathetic nervous system), thereby inhibiting the output of glucose from the liver and improving IR. However, there are currently no specific studies that have elaborated on the detailed signaling pathways of this process [94]. However, Masaki et al. observed that peripheral APN enhanced energy expenditure through an increase in BAT SNS activity and consequent upregulation of BAT UCP‐1 [95]. The regulation of autonomic nerve by central and peripheral pathways is yet to be elucidated. Clinically, higher circulating levels of APN correlate with improved function of both the sympathetic and vagal branches of the cardiac autonomic nervous system in individuals with Type 2 diabetes [96]. On the other hand, SNS physiologically modulates serum adiponectin levels and its synthesis in adipose tissue, suggesting involvement of SNS in adiponectin‐mediated energy homeostasis regulation [97, 98]. In summary, APN is capable of regulating the cross talk between the viscera and the brain via the sympathetic and/or vagal systems, participating in the pathogenesis of metabolic disorders.

APN has been linked to the pathogenesis of cardiovascular disease, diabetes mellitus (DM), and NAFLD [99]. Considering the fact that the liver is one of the most important target organs of APN as well as the central hub in peripheral metabolism, it is not surprising that an abnormal level of APN is associated with NASH/NAFLD and hepatic fibrosis [100]. Serum APN is negatively correlated with NASH/NAFLD and metabolic disorders [100], which could enhance glycolipid metabolism, improve insulin sensitivity, exert anti‐inflammatory, antifibrosis, and antitumor effects [101]. APN regulates liver functions through the following mechanisms: (1) AMPK and PPARα pathway; APN binds to its receptor, which phosphorylates AMPK. Phosphorylated AMPK can then inhibit the transcription of PEPCK, G6Pase, and the phosphorylation of ACC, thus reducing gluconeogenesis and lipid synthesis. This pathway also reduced fibrosis by inhibiting stellate cell activation, transforming growth factor‐β (TGF‐β) induction, and JAK2/STAT pathway activation via leptin. PPARα pathway can play a synergistic role with MAPK pathway to promote beta oxidation, thus preventing liver fibrosis [102, 103]. (2) PI3K/Akt pathway. On activation by APN, PI3K/Akt signaling could improve liver IR [104]. (3) APN can also play an anti‐inflammatory role in inhibiting the release of inflammatory mediators such as interleukin‐1β (IL1‐β) and tumor necrosis factor‐α (TNF‐α) and in blocking the activation of nuclear factor κ B (NF‐κB) [105]. APN could reduce TNF‐α–induced inflammation in the liver of NAFLD mice [106]. It also increases the expression of hepatic IR substrate‐1 (IRS‐1) through the interleukin‐6 (IL‐6)–dependent pathway, thus indirectly improving insulin sensitivity [107, 108] (Table 1) (Figure 2).

APN affects the development of NAFLD in the central and peripheral systems through different mechanisms. In the periphery, it promotes fatty acid oxidation by activating pathways such as AMPK and inhibits the inflammatory response mediated by NF‐κB, effectively delaying the progression of NAFLD from a simple fatty liver to NASH. In the central system, it may exert inhibitory effects on food intake through the autonomic nervous system and regulate energy metabolism to indirectly improve IR and inhibit steatosis. Currently, the mechanism of adiponectin’s effect on NAFLD through adipo–brain–liver cross talk is still in its infancy. More research and elaboration are needed in the future.

## 4. FGF21

FGF21, discovered in 2000, was detected in the adipose tissue, skeletal muscle, and pancreas [109–113]. However, Markan et al. reported that the hepatocyte is the main supplier of circulating FGF21 [114]. Circulating FGF21 plays its role by activating cell‐surface FGF receptors (FGFRS), such as serotypes FGFR1c, 2c, 3c, and 4c [115–117]. These receptors are highly expressed not only in peripheral tissues, such as BAT and WAT, but also in the CNS, such as hypothalamus and hindbrain [118–120]. Recent research has revealed that FGF21 is produced and secreted by tanycytes located in the hypothalamus [121]. It had been reported that Klb is expressed in the hindbrain, specifically within crucial neuronal populations of the DVC, NTS, encompassing the area postrema (AP), as well as in the nodose ganglia housing sensory neurons of the vagal nerve [122]. Besides, conspicuous presence of KLB expression on the cellular membrane of NG neurons and NTS regions was demonstrated as well [123]. Meanwhile, the roles of FGF21 in the neurocontrol of blood pressure functions via the baroreflex afferent pathway (including NTS, NG) had been elucidated [123] (Table 1).

Elevated levels of FGF21 in the bloodstream have been observed under various conditions, including obesity‐related metabolic diseases and NAFLD [124–126]. Numerous studies reported that FGF21 levels were positively correlated with the severity of steatohepatitis, particularly fibrosis, among patients with NASH [127] and individuals with NAFLD [128]. Elevated levels of FGF21 have the potential to impede adipose tissue lipolysis and constrain hepatic lipid accumulation [129, 130], indicating that FGF21 signaling plays an important role in metabolic liver disease.

FGF21 acts as a hormone that exerts multifaceted impacts on glucose and lipid metabolism [131, 132]. (1)MAPK/ERK1/2 pathway: The activation of the receptor complex by FGF21 triggers a series of signaling pathways, which include the phosphorylation of FGFR substrate‐2 and the MAPK cascade. This ultimately leads to the phosphorylation of extracellular signal–regulated kinase1 and 2 (ERK1/2) [116, 133]. (2)AMPK‐SIRT1 pathway: FGF21 triggers the AMPK‐SIRT1 pathway, leading to protein post‐translational modifications [134]. FGF21−/− mice exhibited significantly exacerbated hepatic triglyceride accumulation, which had been associated with increased inflammation and fibrosis. One potential explanation to the observed phenotype in the FGF21 knockout animals could be the disruption in fatty acid activation, along with upregulation of crucial genes involved in the uptake of fatty acids, as well as those in the biosynthesis of diacylglycerol [135, 136]. On the other hand, FGF21 was regulated by PPARα, which controls the transcription of numerous enzymes that facilitate fatty acid oxidation and ketosis [109]. By clarifying how FGFs drive NAFLD progression, our work opens new avenues for treating hepatic disorders [128].

Multiple studies have confirmed the significant roles of FGF21 in regulating lipid and carbohydrate metabolism, as well as maintaining energy and nutrient homeostasis [137]. While the mechanisms behind these effects have been a topic of debate. Studies have shown that FGF21 can directly regulate liver cells or the entire liver [138, 139]. Although other studies have not found that FGF21 has a direct regulatory effect on liver cells, it also exerts indirect regulatory effects through the hypothalamus–pituitary–adrenal (HPA) cortex axis or by activating other peripheral signaling pathways [140]. The above studies indicate that FGF21 has both direct and indirect effects on NAFLD and metabolic liver diseases. Interestingly, FGF21 is present in the CSF in humans, and a linear correlation was observed between serum FGF21 levels and CSF concentrations [141], suggesting that FGF21 could have central effects. Technically, FGF21 could cross the blood–brain barrier and thus is possible to exert the effects on the CNS [122]. Serum FGF21 responded to nutritional stimuli, thereby transmitting information to the brain. Consequently, FGF21 exerts its primary influence on the CNS through its endocrine function [142].

Previous study suggested that FGF21 participates in the maintenance of glucose homeostasis by mediating the cross talk between the liver and brain during prolonged fasting [143]. It enters the brain to directly activate hypothalamic neurons and the HPA axis for the stimulation of hepatic gluconeogenesis [143]. In the subnucleus of the hypothalamus, FGF21 could act on the VMH by reducing the excitability of glucose‐sensitive glutaminergic neurons [144]. Additionally, FGF21 exerts its effects on the ARC by upregulating the expression of AgRP and NPY to induce food intake [145], revealing the role in central energy balance regulation. In the lateral hypothalamic area (LHA), FGF21 activates GABA‐containing neurons expressing dopamine receptor 2 (DRd2) [121, 146]. Moreover, for PVN, the effect of FGF21 was mediated by the β‐klotho receptor complex [147], which is required in weight loss and energy consumption in adipose tissue by activating SNS [145]. A recent article has revealed that FGF21 acts as a messenger between the liver and the brain to reverse NASH: FGF21 directly acts on glutamatergic neurons in the CNS, activating the sympathetic nerve output of the liver, thereby inhibiting the synthesis of new lipids in the liver, reducing the accumulation of liver triglycerides and reversing the fibrotic process; FGF21 binds to the KLB coreceptor on hepatocytes, activating the ERK1/2 signaling pathway, upregulating the expression of cholesterol transport proteins ABCG5/ABCG8, promoting the excretion of cholesterol into bile, thereby reducing the cholesterol content in the liver [140]. FGF21 crosses the blood–brain barrier and activates nesfatin‐1 neurons in the PVN, forming a glucose‐dependent liver–hypothalamic energy regulation axis that coordinates feeding, energy metabolism, and circadian rhythms under physiological conditions. In pathological states, the dysfunction of this axis may be closely related to the development of obesity, metabolic syndrome, and diabetes. Therefore, targeting the FGF21–nesfatin‐1 signaling pathway may provide a new strategy for the treatment of metabolic diseases [148] (Figure 1).

In summary, FGF21 not only modulates the pathogenesis and progression of NAFLD/NASH by regulating glucose and lipid metabolism but also regulates energy balance through the CNS, thereby influencing the pathological process of the disease at multiple levels. In the early stage of NAFLD (simple fatty liver stage), FGF21 can improve IR, promote fatty acid oxidation, and reduce liver lipid accumulation; as the disease progresses to NASH, FGF21 further exerts anti‐inflammatory, antioxidant stress‐inhibiting, and hepatocyte damage‐suppressing effects. Although existing studies suggest that FGF21 has potential protective effects in NAFLD, further research is needed to clarify its combined regulatory network and its clinical translational value as a therapeutic target.

## 5. Nesfatin‐1

Nesfatin‐1 is an 82‐amino‐acid peptide derived from the precursor protein nucleobindin‐2 (NUCB2), first identified by Oh‐I et al. in 2006. NUCB2 is cleaved by prohormone convertases PC1/3 and PC2 into three fragments: nesfatin‐1 (1–82), nesfatin‐2 (85–163), and nesfatin‐3 (166–396). Among these, only nesfatin‐1 inhibits feeding and reduces adiposity following i.c.v. injection [149]. Nesfatin‐1 is an adipokine expressed both in CNS and peripheral tissues. In peripheral tissues, nesfatin‐1 was detected in adipocytes [150], stomach [151], pancreas [150], and liver [152]. In the CNS, NUCB2/nesfatin‐1 was mainly expressed in midbrain (PVN, ARC, LHA, and SON) [149, 153] and hindbrain (DVC, LPBN, NTS, VLM, and LC) [153–156] (Table 1) (Figure 2).

The regulatory functions of nesfatin‐1 in metabolic homeostasis have been investigated for more than a decade [157]. Although the specific receptor of nesfatin‐1 has not been identified yet [158], a previous study from our group had illuminated that growth hormone secretagogue receptor (GHSR) is required for the effects of nesfatin‐1 on glucose metabolism. As GHSR signaling exhibited protective in the progression of NAFLD to liver cancer [159], it suggested that nesfatin‐1 could serve as a promising therapeutic target in metabolic liver diseases. Moreover, our recent work reviewed the central and peripheral functions of nesfatin‐1, extending the potential relationship between nesfatin‐1 and GHSR to neuroprotection and gastrointestinal functions [160]. Although GHSR has been implicated in mediating some of the metabolic effects of nesfatin‐1, current evidence does not conclusively demonstrate that GHSR serves as a direct binding receptor for nesfatin‐1. Rather, the available data suggest that nesfatin‐1 may exert its actions through GHSR‐dependent signaling pathways or via interactions with other transmembrane proteins [161]. Interestingly, nesfatin‐1 could cross the blood–brain barrier in a bidirectional unsaturated manner, suggesting adipose‐derived nesfatin‐1 as a potential contributor to adipo–brain–liver cross talk.

Nesfatin‐1 attenuated lipid accumulation in hepatocytes via an AMPK‐dependent mechanism in vitro, revealing the direct effect of nesfatin‐1 on the liver under metabolic stress [162]. However, whether it could regulate liver metabolism through CNS is still unclear and worth further investigation. Wu et al. found that hypothalamic NUCB2/nesfatin‐1 signaling plays an important role in hepatic metabolism, which might be associated with activation of the mTOR‐STAT3 signaling pathway [163]. Our previous study showed that GHSR mediated the effect of nesfatin‐1 on AKT phosphorylation in the liver of HFD‐fed mice [164]. These results emphasized the value of nesfatin‐1 as an interorgan messenger in metabolic liver diseases.

Central administration of nesfatin‐1 increases the phosphorylation of InsR/IRS‐1/AMPK/Akt/TORC2 and enhances Fos immunoreactivity in hypothalamic nuclei, thereby contributing to glucose homeostasis [165]. The direct neural projections also persist between brain and fat, as well as between brain and liver [166]. NUCB2/nesfatin‐1 immunoreactivity was concentrated in the DVC [153, 154, 156, 165] and sympathetic/parasympathetic preganglionic neuronal populations [154, 165]. The autonomic‐regulating ability of nesfatin‐1 enabled its effects on adipo–brain–liver cross talk.

Using pharmacological and surgical denervation approaches, our recent study revealed that central nesfatin‐1 modulates peripheral lipid metabolism via the hypothalamic–sympathetic nervous system axis [167]. The VMH rich in the sympathetic efferent nerve was identified as a key site for fat browning [27]. Our results suggested that the sympathetic nervous system plays an important role in brain–organ metabolic regulation. Additionally, extracellular signal–regulated kinase (ERK) in PVN had been shown to be a regulatory target of nesfatin‐1. Similarly, the ERK‐dependent sympathetic activation in the hypothalamus is critical for the antiobesity effects of nesfatin‐1 [168]. It is also worth noting that retrograde tracer indicated the presence of neuronal projections from NUCB2/nesfatin‐1–positive neurons to DVA and ARC [169]. Most NUCB2/nesfatin‐1–positive neurons reside in the DMV, projecting to the amygdala [170]. Taken together, the hindbrain structures play important roles in the bidirectional communication between the CNS and the PNS. As early as 2014, our work revealed that nesfatin‐1 modulates the excitability of the glucose‐sensing neurons directly in DVC by electrophysiological recordings in vivo and current‐clamp electrophysiology recordings in vitro, suggesting that nesfatin‐1 might modulates the liver metabolism and energy balance by affecting the neural signal from the DVC to peripheral [171]. Yue et al. identified a fatty acid‐dependent hypothalamic‐DVC neurocircuitry that regulates hepatic lipid metabolism, suggesting that a fatty acid sensing‐dependent hypothalamic‐DVC neurocircuitry might serve as a potential therapeutic target to improve liver metabolism in obesity [172]. Regarding food consumption, the peripheral stimulation of nesfatin‐1 probably resulted in POMC and CART neuron activation and subsequential anorexia independent with leptin, indicating the therapeutic potential of nesfatin‐1 in metabolic diseases under the leptin‐resistant state [173]. It is worth noting that LPBN plays a vital signal delivery role between NTS and the hypothalamus and has well‐qualified direct neural projection to the liver [174]. Furthermore, we have recently investigated the role of LPBN nesfatin‐1 in the regulation of energy balance and glucose‐sensitive neurons [155] in diet‐induced obese mice and further clarified the role of GHSR in this nucleus under metabolic stress [175]. However, whether nesfatin‐1 could regulate NAFLD through LPBN is still unknown and deserves further clarification.

In summary, nesfatin‐1 acts centrally and peripherally to suppress food intake, improve hepatic lipid metabolism, and maintain glucose homeostasis. These beneficial actions highlight its potential as a therapeutic agent for metabolic liver diseases and obesity‐related disorders. Nonetheless, the specific receptor for nesfatin‐1 has not been identified, and its detailed signaling pathways and central neural circuits remain incompletely understood. Further investigation into nesfatin‐1‐mediated neurocircuits may provide novel targets for the pharmacotherapy of metabolic syndrome.

## 6. ANGPTLs

Peptides in the ANGPTLS family share similar structures: a signal sequence located at the N‐terminus, an N‐terminal coiled‐coil domain (CCD), and a C‐terminal fibrinogen‐like domain (FLD). An exception was ANGPTL8, in which the FLD domain is absent [176]. The primary biosynthesis of ANGPTL4, ANGPTL6, and ANGPTL8 occurs primarily in the adipose tissue and liver. Meanwhile, ANGPTL3 is more exclusively synthesized and secreted in the liver (Conklin, 1999) [177, 178]. Although no receptor for ANGPTL8 has been identified, it is widely distributed in WAT, BAT, and brain [179].

Currently, there is preliminary evidence suggesting that liver‐derived ANGPTL3 and ANGPTL8 may be involved in the adipo–brain–liver cross talk. The potential mechanisms include indirectly influencing the lipid metabolism environment throughout the body and acting on the brain, or there may be direct effects on the blood–brain barrier and specific brain regions. However, this latter pathway remains a speculative assumption, and the specific sites of action and signaling pathways are far from being clear. Therefore, this field deserves further in‐depth investigation and exploration in the future. It had recently reported the presence of ANGPTL8 in appetite‐related nuclei, including PVN, DMH, ventromedial hypothalamus, and ARC in the hypothalamus. Both central and peripheral administration of ANGPTL8 could effectively reduce food intake. To sum it up, ANGPTL8 participates in the energy homeostasis regulation via the inhabitation of food uptake via the DMH pathway and regulatory roles in adipose tissue deposits. Intriguingly, ANGPTL8 differentially affected the c‐FOS–positive neurons in different CNS areas that are all supposedly participating in ANGLTL8‐mediated appetite regulation. Further work is guaranteed [180]. Different from the inhibitory effect of ANGPTL3 on peripheral LPL, ANGPTL3 in hypothalamus can activate LPL [181]. The enhanced activity of LPL can promote the lipid uptake of hypothalamic neurons, contributing to increased brain lipid sensing [182, 183] (Figure 2).

In ANGPTLs, ANGPTL3, ANGPTL4, ANGPTL6, and ANGPTL8 are all closely related to liver metabolism. Circulatory dysregulation of glycoprotein homeostasis has been implicated in the pathogenesis of metabolic disorders including diabetes, obesity, and NAFLD (Figure 1). Mechanistic investigations using preclinical models revealed that therapeutic modulation of hepatic ANGPTL3 expression through antisense oligonucleotide (ASO) administration in LDLR−/− mice markedly attenuated hepatic triglyceride accumulation and atherosclerotic plaque formation [184]. Complementary studies demonstrated that peptide‐mediated ANGPTL3 inhibition in ob/ob mice substantially decreased diet‐induced adiposity and hepatic lipid deposition [185], collectively positioning this hepatokine as a pivotal regulator of lipoprotein metabolism with translational potential for NAFLD and cardiovascular disease management (Table 1). While the N‐terminal domain of ANGPTL4 suppresses lipoprotein lipase (LPL) activity, its C‐terminal fragment promotes adipocyte lipolytic processes. Parallel investigations identified ANGPTL6 as a suppressor of hepatic gluconeogenesis through FOXO1‐PI3K‐Akt pathway inhibition [186, 187] (Figure 2). In diabetic models, the ANGPTL8‐mediated regulation of glucose homeostasis was found to operate via PI3K/Akt‐dependent mechanisms [188], with synergistic interaction observed between ANGPTL8 and ANGPTL3/4 in modulating LPL functionality. Notably, ASO‐mediated ANGPTL8 knockdown attenuated high‐fat diet‐induced hepatic steatosis in rodents, potentially through SREBP1‐dependent adipogenic pathway downregulation [189]. This evidence strongly supports the therapeutic potential of ANGPTL8 for metabolic syndromes such as NAFLD.

Beyond its mechanistic role in lipid metabolism, ANGPTL3 has emerged as a clinically relevant therapeutic target. Several ANGPTL3‐directed agents have already entered human studies, and they have demonstrated substantial lipid‐lowering efficacy in patients with severe dyslipidemia. For example, vupanorsen, a hepatically targeted ASO, significantly reduced triglycerides and other atherogenic lipids in Phase 2 studies, whereas evinacumab produced sustained reductions in LDL‐C and was generally well tolerated in longer‐term clinical trials [190, 191]. However, the impact of ANGPTL3 inhibition on NAFLD progression remains uncertain. Notably, vupanorsen was associated with dose‐dependent increases in hepatic fat fraction and liver enzyme elevations, suggesting that hepatic ANGPTL3 suppression may not necessarily translate into benefit for steatosis [192, 193]. In contrast, genetic ANGPTL3 deficiency was not associated with hepatic steatosis in human studies, indicating that pharmacologic effects may differ from lifelong genetic reduction [194]. Therefore, the therapeutic value of ANGPTL3 targeting in NAFLD should be interpreted cautiously, and future trials should incorporate liver‐specific outcomes, including hepatic fat content, inflammation, and fibrosis, to clarify whether systemic lipid lowering can translate into true histological benefit.

In conclusion, the ANGPTL family regulates hepatic lipid and glucose metabolism at the molecular, cellular, and systemic levels. Inhibition or overexpression of the ANGPTL family of adipokines had potential impacts on NAFLD and IR. Thus, hepatokine mimetics or inhibitors could be effective therapeutic approaches for NAFLD and other metabolic diseases. However, the involvement of ANGPTL as an adipokine in organ cross talk between the brain and liver in the regulation of liver disease is still in its infancy. The roles of central ANGPTLs need to be explored in further studies. In the future, the specific central mechanism of action could potentially be studied by injecting ANGPTL into the key nuclei of the hypothalamus.

## 7. Resistin

Belonging to the family of adipokines, resistin is a cysteine‐rich peptide hormone that contains 108 amino acid residues and is rich in cysteine residues encoded by chromosome 19p13.3 [195, 196]. Resistin is mainly synthesized by adipocytes, monocytes, and macrophages from adipose tissue. Notably, there exists a critical species‐specific difference in resistin‐producing cells: Resistin is predominantly secreted by adipocytes in rodents, whereas in humans, its main cellular sources are immune‐related cells including macrophages and monocytes [197]. This distinction largely determines its divergent pathophysiological roles in the adipose–brain–liver axis between rodents and humans, which should be considered when extrapolating preclinical findings to human conditions. It had been reported that circulating resistin concentrations are positively correlated with high‐fat‐diet–induced obesity [196, 198, 199]. As an important factor involved in metabolism, resistin is widely distributed in the liver and brain (Table 1).

In peripheral, resistin can induce hepatocyte steatosis and IR by affecting liver fat metabolism and increasing liver fat content. Benomar et al. reported the resistin‐mediated inhibition of the insulin signaling IRS‐1/PI‐3K/AKT pathway, reducing the activation of protein kinase B and PI3K, ultimately leading to IR in adipocytes [200]. Meanwhile, studies have shown that the administration of resistin neutralizing antibodies in mice resulted in the activation of MAPK and subsequent reduction of liver gluconeogenic enzyme expression levels, glucose levels, and IR [201]. Additionally, there may be a link between resistin and the development of NAFLD [199–201]. In the CNS, the hypothalamus is one of the target tissues of resistin. Central resistin is mainly distributed in the ARC and medial basal part of hypothalamus (MBH). A small amount is also present in the ventrolateral hypothalamic nucleus, ventromedial nucleus, and the dorsal periventricular area [202, 203]. Central resistin regulates the metabolic and endocrine functions of the body by interacting with neuropeptides in the hypothalamus [202–204] (Table 1) (Figure 2).

Central resistin activates hypothalamic NPY neurons, leading to increased expression of inflammatory factors such as SOCS3, TNF‐α, and IL‐6 in the liver, while simultaneously inhibiting the signaling pathways that mediate insulin action (such as decreased phosphorylated Akt and increased G6Pase), thereby increasing glucose production in the liver and exacerbating IR [205]. However, there is still a lack of sufficient research on the intermediate neural pathways from NPY neurons to the liver, and it is possible that they are achieved through autonomic nerves such as the sympathetic nerve. Another related study had shown that the injection of NPY into the ICV of the lateral ventricle can increase resistin mRNA expression levels in WAT [206]. Wilkinson et al. found that a complex network of resistin fibers exists in the ARC of the hypothalamus and extends all the way to the preoptic area [203].

In addition, resistin mRNA was also widely expressed in the anterior and middle lobes of the mouse pituitary gland, and rarely in the posterior lobe [202]. These results suggest that resistin may be produced and secreted from the ARC of the hypothalamus and project into the nerves of the posterior pituitary through the hypothalamic–pituitary tract [202].

Resistin was released de novo in the hypothalamus and hippocampus, suggesting a paracrine effect. It stimulates the release of norepinephrine and dopamine from hypothalamic synaptosomes, further supporting central effects. Modulation of resistin action in the MBH significantly affects hepatic glucose homeostasis and selective inflammatory markers. Central resistin can induce hepatic IR, which is accompanied by the induction of TNF‐α, IL‐6, and SOCS‐3 [204].

Mitochondrial dysfunction is a hallmark of obesity, Type 2 DM, and NAFLD. Wen et al. noted that resistin levels were significantly increased in the liver tissue samples of NAFLD mice, followed by the altered mitochondrial morphology of hepatocytes and IR. Resistin can exacerbate hepatic steatosis in male mice by activating the AMPK/PGC‐1α (peroxisome proliferator–activated receptor‐γ coactivator‐1α[PPARC‐1α]) pathway, leading to a reduction in the mitochondrial content and an increase in lipid accumulation in hepatocytes of high‐fat‐diet–fed animals [199]. Similar studies have shown that resistin inactivates PPARC‐1α and reduces the number of mitochondria by promoting the interaction between PPARC‐1α and P65 in mice. Signal transduction analysis showed that resistin downregulated mitochondrial number through a novel protein kinase C/protein kinase G/P5/PPARC‐1α signaling pathway, further confirming resistin‐mediated mitochondrial dysfunction, exacerbation of hepatic steatosis, and IR induction. Mitochondrial dysfunction seems to be located at upstream, since mitochondrial changes occurred before fat accumulation and IR [207]. These results revealed a novel mitochondrial regulatory pathway and provided new insights into the pathogenesis of NAFLD. In addition, the studies of resistin pointed out a new direction for the treatment of HCC: Resistin promotes the expression of proinflammatory cytokines (such as TNF‐α and IL‐6) and monocyte chemotactic protein in peripheral blood monocytes and macrophages, as well as hepatic stellate cells (HSCs) through NF‐κB. It also activates HSCs and enhances the production of TGF‐β and type I collagen by Kupffer cells to promote fibrosis [208] (Table 1) (Figure 2).

In summary, like other adipokines, resistin secreted in the hypothalamus can also affect hepatic glucose homeostasis and induce hepatic IR. At the same time, resistin plays an important role in liver steatosis, mitochondrial dysfunction, inflammatory response, and IR. However, the relationship between resistin and NAFLD needs to be further explored. One possible mechanism is that resistin induces mitochondrial dysfunction and ROS generation, leading to NASH. More clinical evidence is still needed to support this hypothesis.

## 8. Asprosin

Asprosin is a glucogenic adipokine, which was firstly discovered by Romere et al. in 2016 [209]. Encoded by two exons of FBN1 [210], asprosin is synthesized and released in white adipose tissue during fasting [211]. Asprosin could serve as the ligand for various receptors. Olfactory receptor 4M1(OR4M1) is considered to be the primary receptor for human asprosin. Additionally, olfactory receptor 734 (OLFR734) had also been determined to be one of the receptors of asprosin recently [211] (Table 1).

The actions of asprosin in the center are mediated by specific neurons in different parts of the brain. They activate sympathetic nerves to exert indirect regulatory effects on peripheral organs [212]. It had been reported that asprosin could lower the glucose release level in the liver and is beneficial to Type II diabetes and metabolic syndrome therapeutically [210]. Nonetheless, asprosin has appetite‐stimulating effects and may contribute to IR [213].

In the periphery, asprosin promotes the release of glucose in the liver and stimulates the appetite of hypothalamus by activating the G protein‐cAMP‐PKA pathway in the liver [214]. Asprosin is released from white adipose tissue and then transported to the liver and binds to the OLFR734 [215]. Then, G protein–coupled receptor (GPCR) activates the adenylyl cyclase–PKA–cAMP‐responsive element binding (CREB) pathway, promoting glucose production and release [216]. The glycogenic effects of asprosin are also mediated through the activation of PKA signaling [214]. Central asprosin has the effect of promoting food intake. Within the ARC, asprosin exerts direct regulatory effects on orexigenic AgRP neurons through the activation of the G protein‐coupled cAMP‐PKA signaling cascade. Notably, these neurons exhibit abundant expression of protein tyrosine phosphatase receptor type D (Ptprd), a membrane‐bound receptor demonstrating high ligand specificity for asprosin. Clinical evidence reveals that conditional knockout of Ptprd in AgRP neuronal populations leads to marked attenuation of metabolic parameters including appetite stimulation, glycemic control, and adiposity regulation, establishing Ptprd as the primary receptor mediating asprosin’s orexigenic effects. Meanwhile, by inhibiting the adjacent POMC^+^ neurons, a “double blow” is formed to strongly stimulate appetite. Additionally, asprosin can pass through the blood–brain barrier and enter the cerebrospinal fluid, directly acting on the feeding center of the hypothalamus. Long‐term excessive eating and obesity are the core driving forces for NAFLD. Therefore, central asprosin indirectly increases the risk of lipid deposition, IR, inflammation, and fibrosis in the liver by promoting obesity [217] (Table 1) (Figure 2).

Notably, asprosin hormone is closely related to NAFLD. Clinical evidence reveals a strong correlation between circulating asprosin levels and the pathophysiology of NAFLD in obese pediatric patients. Plasma asprosin levels were significantly higher in obese children with NAFLD compared to both obese non‐NAFLD peers and lean controls [218]. Similar results have been shown in adults with NAFLD [219]. Hypothalamic asprosin modulates two key nuclei—ARC and PVN—inducing appetite stimulation and sympathoexcitatory responses, respectively (Figure 2). Peripherally, this adipokine impairs insulin signaling pathways, exacerbating IR, inflammatory cascades, and endoplasmic reticulum stress (Figure 1). Clinical correlations further confirm its positive association with metabolic dysregulation including obesity and cholesterol imbalance. Collectively, these findings establish asprosin as a critical regulator of systemic metabolism, highlighting its therapeutic potential for metabolic disorders. However, precise mechanistic delineation of its context‐dependent physiological versus pathological roles requires further exploration.

## 9. Conclusions

NAFLD is an important disease of liver metabolism. Fatty acid accumulation, IR, and mitochondrial dysfunction are key factors in NAFLD and are strongly associated with the regulation of adipokines secretion. In the present review, we synthesize the mechanisms and transduction pathways of seven adipokines in the liver and brain and systematically review the role of adipokines in liver disease. Central adipokines, such as leptin, exert their effects through sympathetic/parasympathetic signaling to the liver. However, more adipokines have independent functions in the central and peripheral areas, and there are few studies in the field of direct dialog between the liver and brain. Research on adipokines on the regulation of liver diseases is still in its infancy, which provides a new direction for future research.

It is essential to consider the integration of adipokine signaling with gut‐derived hormones (such as GLP‐1 and ghrelin) within key central nuclei like the DVC and ARC. These regions serve as critical hubs not only for adipokine processing but also for gut hormone signaling, where convergent inputs likely generate synergistic or antagonistic effects on hepatic energy balance [220, 221]. Elucidating how leptin, adiponectin, and resistin interact with anorexigenic (GLP‐1) and orexigenic (ghrelin) gut signals at the neuronal circuit level will provide a more comprehensive understanding of the brain’s regulatory network governing NAFLD progression, thus providing new directions for future research.

**Nomenclature**
AgRP: agouti‐related peptide
Akt: protein kinase B
AMPK: adenosine monophosphate–activated protein kinase
ANGPTL: angiopoietin‐like protein
AP: area postrema
APN: adiponectin
ARC: arcuate nucleus
BAT: brown adipose tissue
cAMP: cyclic adenosine monophosphate
CART: cocaine‐ and amphetamine‐regulated transcript protein
CCD: coiled‐coil domain
CNS: central nervous system
Cre: cyclization recombination enzyme
CREB: cAMP‐responsive element binding
CSF: cerebrospinal fluid
DM: diabetes mellitus
DMH: dorsomedial hypothalamus
DMN: dorsal motor nucleus
DRd2: dopamine receptor 2
DVC: dorsal vagal complex
ERK: extracellular regulated protein kinases
FBN1: fibrillin 1
FFA: free fatty acid
FGF21: fibroblast growth factor 21
FGFR: fibroblast growth factor receptor
FLD: fibrinogen‐like domain
FOXO1: forkhead box protein O1
GHSR: growth hormone secretagogue receptor
GPCR: G protein–coupled receptor
HCC: hepatocellular carcinoma
HFD: high‐fat diet
HPA: hypothalamic–pituitary–adrenal
HSCs: hepatic stellate cells
IL1‐β: interleukin 1‐β
IL‐6: interleukin‐6
InsR: insulin receptor
IR: insulin resistance
IRS: insulin receptor substrate
JAK2: Janus kinase 2
KLB: Klotho beta
LC: locus coeruleus
LEP‐Rs: leptin receptors
LHA: lateral hypothalamic area
α‐MSH: α‐melanocortin‐releasing hormone
LPBN: lateral parabrachial nucleus
LPL: lipoprotein lipase
MAPK: mitogen‐activated protein kinase
MBH: medial basal nucleus of the hypothalamus
MC4R: melanocortin receptor 4
MSG: monosodium glutamate
NAFLD: nonalcoholic fatty liver disease
NASH: nonalcoholic steatohepatitis
NF‐κB: nuclear factor‐κB
NG: nodose ganglion
NPY: neuropeptide Y
NTS: nucleus tractus solitarius
NUCB2: nucleobindin‐2
OLFR734: olfactory receptor 734
OR4M1: olfactory receptor 4M1
P65: nuclear factor kappa‐light‐chain‐enhancer of activated B cell p65 subunit
PC: prohormone/proprotein convertase
PDE3: phosphodiesterase 3B
PGC‐1α/PPARC‐1α: peroxisome proliferator–activated receptor‐γ coactivator‐1α
PI3K: phosphatidylinositol 3‐kinase
PKA: protein kinase A
POMC: proopiomelanocortin
PPAR‐α: peroxisome proliferator activating receptor‐α
Ptprd: protein tyrosine phosphatase receptor δ
PVN: paraventricular nucleus
ROS: reactive oxygen species
SHP2: protein tyrosine phosphatase nonreceptor type 11
SIRT1: sirtuin1
SNS: sympathetic nervous system
SOCS‐3: suppressors of cytokine signaling
SON: supraoptic nucleus
SREBP1: sterol‐regulatory element binding protein 1
STAT3: signal transducer and activator of transcription 3
T2DM: Type 2 diabetes mellitus
TG: triglyceride
TGF‐β: transforming growth factor‐β
TNF‐α: tumor necrosis factor‐α
TORC: target of rapamycin protein complex
UCP‐1: uncoupling protein 1
VLDL: very‐low‐density lipoprotein
VLM: ventrolateral medulla
VMH: ventromedial hypothalamus
WAT: white adipose tissue

## Author Contributions

Yule Su, Yiping Yang, Wenjuan Sun, and Junhua Yuan contributed to conceptualization; Yule Su, Yiping Yang, Wenjuan Sun, Zhouxi Yu, Yilin Wang, Shixin Liu, Ziyi Chen, Yanfei Li, Xi Yang, Junyan Liu, Caikun Han, and Yaqin Wang contributed to original draft preparation; Junhua Yuan and Yaqin Wang contributed to funding acquisition. Junhua Yuan contributed to visualization, supervision, and project administration.

## Funding

This work was supported by Shandong Postdoctoral Science Foundation (No. SDCX‐ZG‐202400024), Qingdao Postdoctoral Researchers Applied Research Program (No. QDBSH20240102167), National Natural Science Foundation of China (Nos. 82100854 and 82200774), Shandong Provincial Natural Science Foundation, China (No. ZR2024QH261), and National College Students Innovation and Entrepreneurship Training Program of China (No. X2025110650750).

## Disclosure

All authors have read and agreed to the published version of the manuscript.

## Conflicts of Interest

The authors declare no conflicts of interest.

## Supporting Information

Additional supporting information can be found online in the Supporting Information section.

## Supporting information


**Supporting Information** The following supporting information are available online: Figure S1. The neural projections of the central nuclei.

## Data Availability

Data sharing is not applicable to this article as no datasets were generated or analyzed during the current study.
