# Supplementary material for: Adipokine in Metabolic Liver Disease: Adipo–Brain–Liver Cross talk
Source: Int J Endocrinol. 2026 Aug 5;2026:9009628. doi: 10.1155/ije/9009628 (PMC13443135; doi:10.1155/ije/9009628)
Supplement: Supplementary file 1 [file IJE-2026-9009628-s001.zip › Figure S1.pdf]

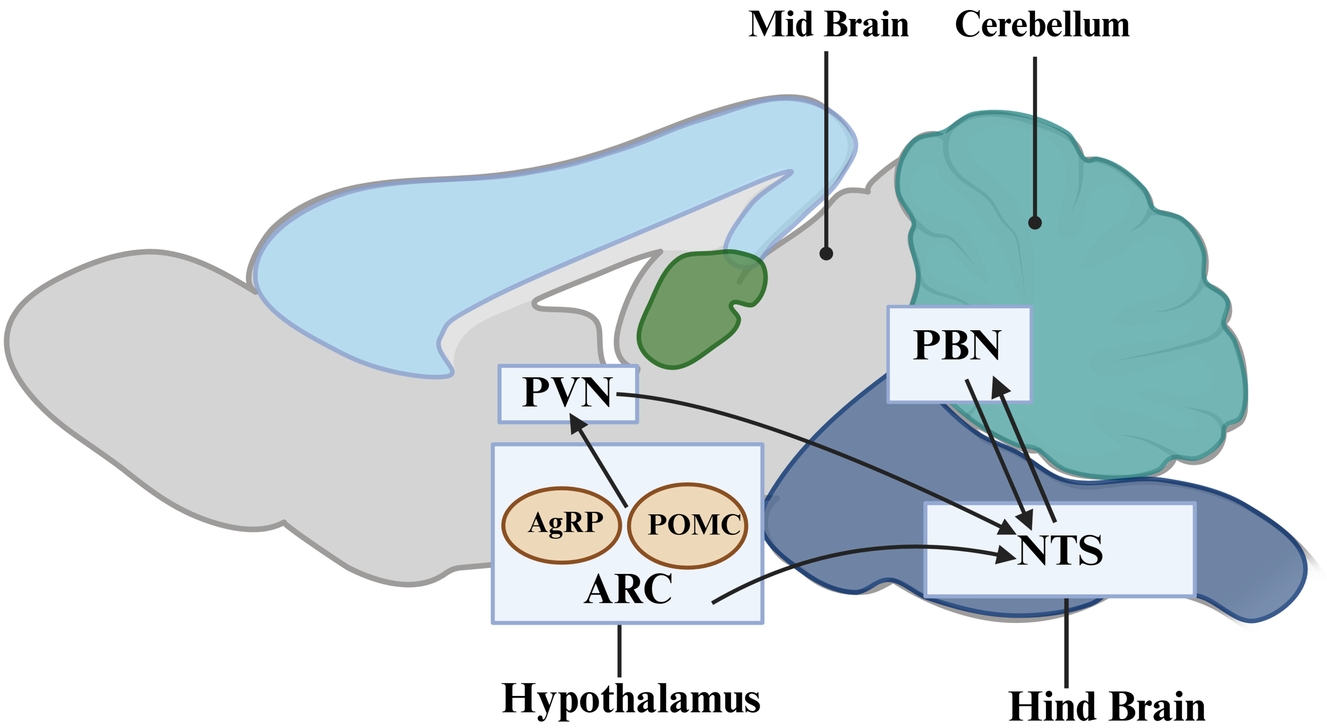
Mid Brain
Cerebellum
PBN
PVN
AgRP
POMC
ARC
NTS
Hypothalamus
Hind Brain
