# Supplementary material for: Adipokine in Metabolic Liver Disease: Adipo–Brain–Liver Cross talk
Source: Int J Endocrinol. 2026 Aug 5;2026:9009628. doi: 10.1155/ije/9009628 (PMC13443135; doi:10.1155/ije/9009628)
Supplement: Supplementary file 1 [file IJE-2026-9009628-s001.zip › supplementary_material 260308.docx]

**Figure S1. The neural projections of the central nuclei.** After the ARC in the hypothalamus perceives the signals from peripheral adipokines, it sends neural projections to the PVN through AgRP and POMC neurons. At the same time, both of them can also transmit information to the NTS to send signals to the periphery. In PBN, LPBN participates in the regulation of appetite and energy balance through bidirectional regulation with NTS, thereby indirectly influencing liver function.
